# Post-treatment with the PPAR-γ agonist pioglitazone inhibits inflammation and bacterial growth during *Klebsiella* pneumonia

**DOI:** 10.1186/s12931-021-01823-8

**Published:** 2021-08-19

**Authors:** Ivan Ramirez-Moral, Bianca Lima Ferreira, Alex F. de Vos, Tom van der Poll

**Affiliations:** 1 Center of Experimental and Molecular Medicine, Amsterdam UMC, University of Amsterdam, Amsterdam, The Netherlands; 2 Amsterdam Infection & Immunity Institute, Amsterdam, The Netherlands; 3 Division of Infectious Diseases, Department of Medicine, Escola Paulista de Medicina, Universidade Federal de Sao Paulo, Sao Paulo, Brazil; 4 Division of Infectious Diseases, Amsterdam UMC, University of Amsterdam, Amsterdam, The Netherlands; 5 Amsterdam UMC, Location Academic Medical Center, University of Amsterdam, Meibergdreef 9, Room G2-130, 1105 AZ Amsterdam, The Netherlands

**Keywords:** PPAR-γ, Pioglitazone, Pneumonia, *Klebsiella*

## Abstract

Agonists of peroxisome proliferator-activated receptor (PPAR)-γ have been suggested as potential adjuvant therapy in bacterial pneumonia because of their capacity to inhibit inflammation and enhance bacterial clearance. Previous studies only assessed the effects of pretreatment with these compounds, thereby bearing less relevance for the clinical scenario. Moreover, PPAR-γ agonists have not been studied in pneumonia caused by *Klebsiella pneumoniae*, a common human respiratory pathogen of which antibiotic treatment is hampered by increasing antimicrobial resistance. Here we show that administration of the PPAR-γ agonist pioglitazone 6 or 8 h after infection of mice with a highly virulent strain of *Klebsiella pneumoniae* via the airways results in reduced cytokine and myeloperoxidase levels in the lungs at 24 h after infection, as well as reduced bacterial growth in the lungs and decreased dissemination to distant organs at 42 h post-infection. These results suggest that pioglitazone may be an interesting agent in the treatment of *Klebsiella* pneumonia.

**To the editor,**

Peroxisome proliferator-activated receptor (PPAR)-γ is a transcription factor belonging to the PPAR subfamily of nuclear hormone receptors [1]. While originally studied for its role in lipid and glucose metabolism, by now the role of PPAR-γ in the regulation of immune responses has been widely recognized [1, 2]. Endogenous ligands that can stimulate PPAR-γ activity include fatty acids and eicosanoids. Considering the broad effects resulting from PPAR-γ activation several synthetic agonists have been developed, in particular thiazolidinediones such as pioglitazone, rosiglitazone, troglitazone, and ciglitazone [1]. Amongst others, these PPAR-γ agonists have been studied as a potential adjunctive therapeutic strategy in bacterial infections [2].

Pneumonia is a major cause of morbidity and mortality worldwide [3]. Treatment of pneumonia by antimicrobial agents has become more difficult due to the emergence of multidrug resistant pathogens. Antimicrobial resistance is especially problematic in infections caused by gram-negative bacteria such as *Pseudomonas (P.) aeruginosa* and microorganisms belonging to the Enterobacteriaceae family, such as *Klebsiella (K.) pneumoniae* [4, 5]. Previous research on the potential of PPAR-γ agonists in gram-negative pneumonia has focused on *P. aeruginosa* [6]*.* PPARγ activation enhanced phagocytosis and killing of *Pseudomonas* by macrophages, and pretreatment with pioglitazone resulted in lower bacterial counts in lung homogenates of mice infected with this bacterium via the airways [7]. With regard to gram-positive pneumonia, our group showed that pretreatment with ciglitazone lessens *Streptococcus (S.) pneumoniae*-induced lung inflammation in mice by reducing bacterial outgrowth and proinflammatory cytokine production [8]. Of note, however, rosiglitazone treatment compromised bacterial clearance during post-influenza super-infection by *Staphylococcus aureus* [9]. To the best of our knowledge the effect of PPAR-γ stimulation during *Klebsiella* pneumonia in vivo has not been studied. In vitro, troglitazone and rosiglitazone were shown to increase the ability of rat alveolar macrophages to phagocytose opsonized *K. pneumoniae* [10]. In the present study we aimed to study the effect of pioglitazone posttreatment, (i.e., administered during an ongoing infection) in pneumonia caused by a highly virulent *K. pneumoniae* strain.

Pneumonia was induced in female C57BL/6 mice (Charles River, Maastricht, the Netherlands; 8–10 weeks of age) by intranasal inoculation of *K. pneumoniae* serotype 2 (43816; ATCC, Rockville, MD; 10^4^ colony‐forming units, CFU) as previously described [11–13]. Bacterial counts were determined in organ homogenates and blood as described [11–13]. Tumor necrosis factor (TNF)-α, interleukin (IL)-6, IL-1β, IL-10, C-X-C motif ligand (CXCL)2 and myeloperoxidase (MPO) were measured in lung homogenates by ELISA according to manufacturer’s instructions (R&D Systems, Minneapolis, MN, USA). Statistical analysis was done using GraphPad Prism 7.03. The number of mice and the statistical tests used for each data set are described in the figure legends. A *P* value < 0.05 was considered statistically significant.

Two treatment strategies with pioglitazone (administered by intraperitoneal injection) were evaluated. In a first experiment mice were infected with *K. pneumoniae* via the airways and treated with a single dose of pioglitazone (20 mg/g body weight; Cayman Chemical, Ann Arbor, MI, USA) or vehicle (10% dimethylsulfoxide in PBS) intraperitoneally 8 h later. At 24 h after infection all mice showed high bacterial loads in the lungs, which were not different between treatment groups; likewise, bacterial burdens in distant body sites (blood, spleen and liver) were similar in pioglitazone and vehicle treated mice (Fig. 1A). Pioglitazone strongly reduced TNF, IL-6, IL-1β, CXCL2 and MPO concentrations in whole lung homogenates (Fig. 1B). We next studied the longer-term consequences of pioglitazone treatment in an experiment in which pioglitazone (10 mg/g) or vehicle was given at 6 and 30 h after infection and effects were assessed 42 h post-infection. In this setting, pioglitazone reduced bacterial loads in lungs and distant organs (Fig. 2A) and inflammatory mediators and MPO levels in lungs, although the difference between groups did not reach statistical significance for IL-6 and IL-1β (Fig. 2B).

**Fig. 1:**
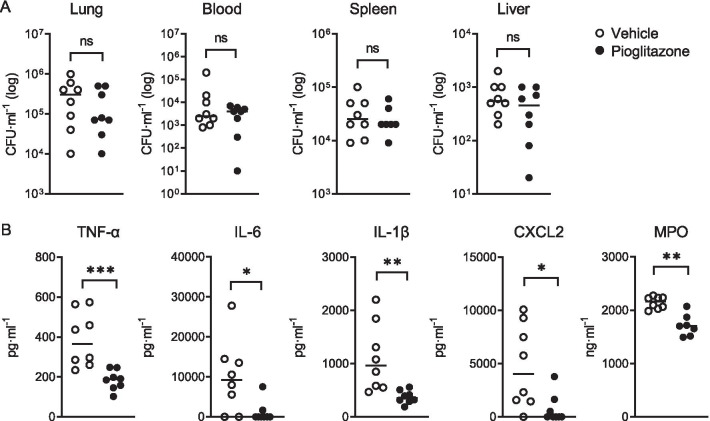
Short-term effect of pioglitazone in *Klebsiella*-induced pneumonia. Mice were infected with *K. pneumoniae* via the airways and treated with a single dose of pioglitazone (20 mg/g body weight) or vehicle intraperitoneally (n = 8 per group) 8 h later; measurements were done 24 h after infection. **A** Bacterial loads in lungs, blood, spleen and liver. **B** TNF, IL-6, IL-1β, CXCL2 and MPO levels in lung homogenates. Graphs show median and every dot represents one individual mouse. P values were calculated using Mann–Whitney *U* tests. *P < 0.05, **P < 0.01, ***P < 0.001, *ns* not significant

**Fig. 2:**
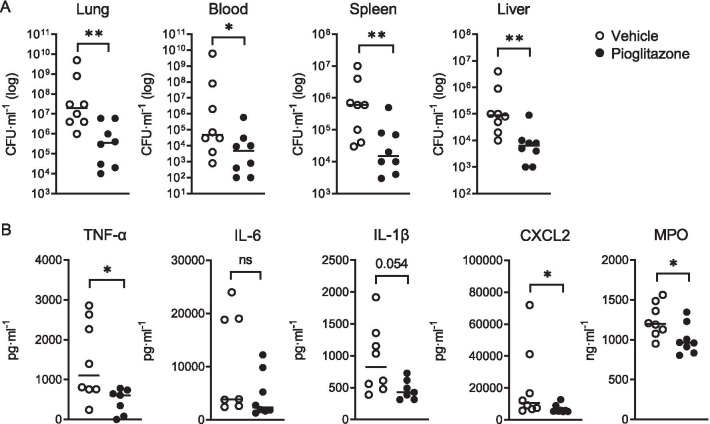
Influence of pioglitazone administration 42 h after induction of pneumonia. Mice were infected with *K. pneumoniae* via the airways and treated with pioglitazone (10 mg/g) or vehicle at 6 and 30 h after infection (n = 8 per group); effects were assessed 42 h post-infection. **A** Bacterial loads in lungs, blood, spleen and liver. **B** TNF, IL-6, IL-1β, CXCL2 and MPO levels in lung homogenates. Graphs show median and every dot represents one individual mouse. P values were calculated using Mann–Whitney *U* tests. *P < 0.05, **P < 0.01, ***P < 0.001, *ns* not significant

The present results of pioglitazone mediated inhibition of inflammatory responses in the lungs during *Klebsiella* pneumonia is in agreement with the previously described effects of ciglitazone during pneumonia caused by *S. pneumoniae*-induced pneumonia [8]. PPAR-γ agonists can exert anti-inflammatory effects on macrophages, including inhibition of proinflammatory cytokine production, via multiple mechanisms, encompassing inhibition of nuclear factor-ĸB and mitogen-activated protein kinases [2, 6]. The role of PPAR-γ in the anti-inflammatory potential of macrophages is further supported by the finding that disruption of endogenous PPAR-γ in myeloid cells impairs alternative macrophage activation [14], and is associated with a spontaneous low-grade inflammatory response in lungs of mice [15]. In pneumonia caused by *S. pneumoniae* mice with macrophage-specific PPAR-γ deficiency displayed impaired antibacterial defense [15]. Together these data suggest that the inhibitory effect of both endogenous PPAR-γ and synthetic PPAR-γ agonists like thiazolidinediones on inflammation in the lungs drives its bacterial growth limiting effect during pneumonia.

PPAR-γ agonists have been extensively studied in the context of lung inflammation [6, 16]. Previous studies have reported on the effects of pre-treatment with a thiazolidinedione in mouse models of *Pseudomonas* and pneumococcal pneumonia [6–8], and bacterial superinfection following influenza [9]. The present study is the first to describe the effect of a PPAR-γ agonist during pneumonia caused by *K. pneumoniae*, a common human respiratory pathogen for which adjunctive therapies are urgently needed [5]. Moreover, this investigation is the first to investigate the effect of administration of a PPAR-γ agonist in mice with an ongoing lung infection, thereby more closely mimicking a clinical scenario. Our results argue for clinical evaluation of PPAR-γ agonists as immune modulating agents in the treatment of bacterial pneumonia.

## Data Availability

Not applicable.
